# COVID-2019 is still rapidly spreading in Iran; isn’t it the time to call for international action?

**DOI:** 10.34172/hpp.2020.16

**Published:** 2020-03-19

**Authors:** Haidar Nadrian

**Affiliations:** ^1^Associate Editor, Health Promotion Perspectives; ^2^Assistant Professor, Ph.D. in Health Education and Promotion, Social Determinants of Health Research Center, Tabriz University of Medical Sciences, Tabriz, Iran

In the Islamic Republic of Iran, the first 43 cases of the Coronavirus Disease 2019 (COVID-19) with eight deaths were reported from February 19 to 23, 2020.^1^ By the time of writing this paper, 1501 cases with 66 deaths are reported.^2^ Day-by-day, the virus is getting spread throughout the country and the world, and we are all witnessing an escalating trend of COVID-19 morbidity and mortality in the world and Iran, in particular. One may compare the trends between affected countries and Iran and find no difference in the trends between the countries. But, with a more focus on the situation of Iran several issues may be identified, which may not be seen in many other countries. First of all, a number of exported cases originated from Iran is being reported, which suggests “an underlying burden of disease in the country than is indicated by reported cases”^1^. Second, estimating the mortality rates for affected countries,^2^ Iran’s mortality rate for the disease seems to be higher than those in other countries. Third, the detection rate in Iran seems to be low, and fourth, access to disinfectants, masks, and sanitizers as well as antiviral drugs with indications to be used against COVID-19 seems to be limited in the country. Well, what do all these public health issues in Iran mean? What may be the reasons for such problems?

Answering the first question, it means that with such a progress of the virus in Iran, a large epidemic could further fuel widespread dissemination of COVID-19^1^ not only in Iran, but also throughout the world. To answer the second question, one may bear in mind the role of international sanctions. Such a critical situation have to be considered as a meta-political issue, within which all political limitations against Iran that may seemingly deteriorate the crisis in the country have to be bypassed. In the current situation of Iran, there are some realities that are not deniable; Yes, detection rate seems to be low, medication therapy is seemingly inadequate, and as a consequence, mortality rate and the number of exported cases originated from Iran are high.^3^ But, why? Who care about? Is it a national issue or an international concern? When there are not enough kits to diagnose the cases, and when there are not enough antiviral drugs to cure the patients with, what should the Iranian health system do?

Here, the role of international organizations and policymakers at an international level is highlighted. Of course, some efforts have been made to help Iran fighting the disease. In February 28, World Health Organization (WHO) dispatched the fifth batch of testing kits to Iran,^4^ and in February 29, a new pack of humanitarian aid from the group of Chinese medical experts was entered into the country.^4^ But, as previously suggested, more actions are needed^5^ to overcome the challenges. Although humanitarian products are legally allowed to be sold to Iran, Iranian companies have found it difficult to process the payments with banks that are overwhelmingly unwilling to risk sanctions by trading with the country.^6^ So, isn’t it a time to emergently find a bypass for the sanctions? Making international commitment by politicians and governments has been recommended^5^ as a global strategy to control the disease. Organizations like WHO, World Trade Organization (WTO), United Nations World Tourism Organization (UNWTO), and the United Nations Children’s Fund (UNICEF) are needed to make much more timely and effective efforts to address the issue in Iran. Active role of international charities and other non-governmental organizations at national and international levels are also emphasized. In the case of not attempting to help Iran to control the disease in a more timely and effective manner, and with consideration on the high likelihood of outward dissemination of the epidemic to the less developed countries with poor capacity to respond to the epidemic,^1^ an international human disaster may not be so unexpected

## Ethical approval

Not applicable.

## Competing interests

The author declares that he has no competing interests.

## Authors’ contributions

HN wrote the initial draft. He made revisions to develop a second draft and provided additional edits to produce the final version, and finally reviewed and approved the final letter prior to submission.
